# A Manual of Surgical Treatment

**Published:** 1912-06

**Authors:** 


**A Manual of Surgical Treatment.** By Sir W. WATSON CHEYNE, Bart., C.B., and F. F. BURGHARD, M.S. New Edition, entirely revised and largely re-written with the assistance of T. P. LEGG, M.S., F.R.C.S., and ARTHUR EDMUNDS, M.S., F.R.C.S. In five vols. Vol. I, pp. xxii, 552; vol. II, pp. xxviii, 570. London: Longmans, Green & Co. 1912.

This work has already received such a well-merited and world-wide recognition that we gladly welcome the new and revised edition, in which the assistance of Legg and Edmunds has been received by the original authors.

The volume deals with general surgical diseases, wounds and their complications, infective diseases, tumours and deformities. It also contains an appendix by Dr. Silk on the administration of anæsthetics, and one by Dr. Emery on the examination of the blood.

The great value of the manual consists in the fact that it records the personal experiences and convictions of two of the most eminent of English surgeons. The details of treatment are described with a clearness and precision which leaves nothing to be desired. It is, however, a matter of regret that in a new edition so much that is old-fashioned should still find a prominent place. This is particularly the case in the present volume dealing with general principles of asepsis and antisepsis. As examples, the following may be mentioned. The iodine method of catgut preparation is omitted; the skin is to be prepared for operations by the long and tedious chemical processes, and the iodine method, in spite of the great amount of work that has proved its excellence is dismissed as having no advantages over the old method with its six-fold change of solutions. The surgeon is depicted as prepared for operations with a short-sleeved gown, the hairy arms being exposed for contact with instruments or ligatures. The marine sponge is greatly extolled over the simple swab. Clean wounds are to be bathed with strong chemical solutions during the process of dressing. In the treatment of talipes the value of direct attack upon the bones, *e.g.* the astragalus, is considered as only suitable for the most advanced and intractable cases.

The book will, however, in spite of these drawbacks, maintain its high position for purposes of reference.

Vol. II deals with the surgical affections of the different tissues considered separately—first of the soft tissues, then of the bones, and concludes with a systematic account of various amputations. In almost every chapter new matter and new illustrations have been added, which bring the treatise well up to date. Some of the newer sections, which are specially good, are the following:—Treatment of injured nerves and different varieties of nerve suture, the indications for and technique of the excision of the posterior nerve roots, the suture of arteries, and the details of Matas' operations for aneurysms; and an excellent discussion of the various methods of treating fractures and their complications, the more recent operative measures being described.

In the other parts the original standard of excellence has been maintained or improved upon in such a way as to fully retain the position of this work as the standard one for the purposes of reference.

